# The sovereign money initiative in Switzerland: an economic assessment

**DOI:** 10.1186/s41937-017-0010-y

**Published:** 2018-01-25

**Authors:** Philippe Bacchetta

**Affiliations:** University of Lausanne, Swiss Finance Institute, CEPR, Lausanne, Switzerland

## Abstract

The sovereign money initiative will be submitted to the Swiss people in 2018. This paper reviews the arguments behind the initiative and discusses its potential impact. I argue that several arguments are inconsistent with empirical evidence or with economic logic. In particular, controlling sight deposits neither stabilizes credit nor avoids financial crises. Also, assuming that deposits at the central bank are not a liability has implications for fiscal and monetary policy, and Benes and Kumhof (The Chicago Plan Revisited, 2012) do not provide support for the reform as they do not analyze the proposed Swiss monetary reform and their closed-economy model does not fit the Swiss economy. Then, using a simple model with monopolistically competitive banks, the paper assesses quantitatively the impact of removing sight deposits from commercial banks’ balance sheets. Even though there is a gain for the state, the overall impact is negative, especially because depositors would face a negative return. Moreover, the initiative goes much beyond what would be the equivalent of full reserve requirement and would impose severe constraints on monetary policy; it would weaken financial stability rather than reinforce it; and it would threaten the trust in the Swiss monetary system. Finally, there is high uncertainty both on the details of the reform and on its impact.

## Background

The Swiss people will vote in 2018 on an initiative for monetary reform. The proposal is to have *sovereign money*, where only the Swiss National Bank (SNB) can issue money and where money includes banknotes and scriptural money from non-banks.^1^ In principle, scriptural money means sight deposits included in M1. The reform would imply that all sight deposits in Swiss francs would be transferred outside commercial banks’ balance sheets and would be deposited at the SNB. The SNB would control the quantity of these sight deposits. The initiative also proposes that the SNB distributes funds to the state or directly to households. These funds would come from the existing sight deposits the SNB receives and from new money creation.

The objective of this paper is twofold. First, it reviews the main arguments behind the reform, and second, it discusses the potential impact of its implementation on the Swiss economy.^2^ The perspective taken in the paper is the one of an academic and of a macroeconomist. As a macroeconomist, I would like to put the reform in the perspective of current knowledge in the field. As an academic, I would like to examine the intellectual rigor of the arguments. From both perspectives, this review will be critical. First, even though it is a reform of macroeconomic nature, the motivation behind the initiative fundamentally ignores most of the existing literature in macroeconomics. Second, the arguments are often vague and incomplete and sometimes misleading or incorrect.

The sovereign money reform is obviously related to the proposals for full reserve banking and to the “Chicago plan,” where commercial banks are imposed a 100% reserve requirement on deposits. Sovereign money is similar to full reserve coverage, but it goes one step further as it gives full control of sight deposits by the central bank.^3^ Moreover, the initiative goes much further than the concept of sovereign money. It would introduce constraints on monetary policy and would imply that SNB liabilities are no longer matched by its assets. It would also impose restrictions on minimum holding periods for non-monetary financial assets such as savings deposits.

A major feature of the sovereign money reform is that money would not bear any interest. This implies that there would never be interest on checking accounts, even in periods of high interest rates. This means that the reform would increase the cost of holding money. As pointed out by Friedman (1969), holding money is in general costly, because it bears a return below the one on other assets, and this opportunity cost should be minimized. Instead, the Swiss sovereign money reform would increase this cost.

While the idea of full reserve requirements has received some attention in the literature,^4^ it is difficult to find much literature on sovereign money. Consequently, the proponents of the initiative often refer to studies on full reserve requirements, even though they insist that sovereign money is different from reserve requirements. However, the references mentioned do not examine all the aspects included in the initiative and do not consider the Swiss case. For example, the paper by Benes and Kumhof (2012) is often cited in support of the initiative, but it does not consider the same policy experiment. Moreover, as explained in the “The arguments behind the initiative” section, the model of Benes and Kumhof does not fit the Swiss economy.^5^ Advocates of sovereign money also refer to heterodox views of monetary economics, for example, a literature labeled as Post Keynesian monetary economics or Modern Monetary Theory. However, authors in this literature reject the arguments behind sovereign money reform (e.g., see Fontana and Sawyer, 2016, 2017, and Nersisyan and Wray, 2016).

The idea of sovereign money is actually based on a manifesto written by Huber and Robertson (2000), henceforth HR. These two authors do not relate their arguments to the existing literature in monetary economics. Even though the motivation for monetary reform is not totally clear, they provide several arguments behind their proposal, some of which I will review in the next section.^6^ At this stage, it is interesting to notice that the original sovereign money proposal by HR preceded the global financial crisis, so that avoiding crises was not its main motivation.

Even though some of the arguments are not fully explicit, there are several hidden assumptions that run counter to our current knowledge in macroeconomics. In particular, a major argument behind the sovereign money proposal is that controlling money allows the stabilization of credit.^7^ This in turn will help stabilize the business cycle. If this is left to commercial banks, HR write: “They expand credit creation in upswings, and reduce it in downswings. The result is that bank-created money positively contributes to overheating and overcooling business cycles, amplifying their peaks and troughs,...(p. 37)”*.* However, HR provide no evidence for their claim. While their first sentence is correct, there are two fundamental problems with their second sentence. First, there is little empirical evidence that money amplifies business cycles in modern economies. On the contrary, bank deposits tend to decline before financial crises (see Jordá et al., 2017). Second, the link between money and credit is weak. As I discuss below, there is no correlation between changes in money and changes in credit in Switzerland. Looking at developed economies, Schularick and Taylor (2012) show there was a close link between credit and broad money before World War II, but there has been a decoupling after World War II. Schularick and Taylor also discuss the distinction between the “money view” and the “credit view” in macroeconomics. The defenders of sovereign money clearly worry about credit, but they want to control it by controlling money. This perspective is inconsistent with empirical evidence.

The arguments in favor of the reform are also often backward looking, citing facts, or reasonings in the nineteenth century or early twentieth century. But the role of money used for transactions has clearly changed in the last decades. It is likely to keep changing in the near future, and the liquidity services of demand deposits will most likely drop.^8^ With a decline in the demand for transaction money, the potential revenue for the central bank, one of the main arguments for reform, would also shrink. The development of new forms of e-money will also require a different analysis. However, at this stage, we ignore what form of e-money will be widely used. An important question is whether central banks will issue e-currency in the future. Here, we need to distinguish between two different cases. First, central banks could offer e-currency directly to non-banks in addition to the existing system. This is the option currently considered by some central banks. It remains to be seen if there would be a demand for such a product.^9^ The second case is where e-currency would replace all sight deposits, i.e., it would be compulsory to use central bank e-money instead of sight deposits at commercial banks. The latter system would be similar to a sovereign money system.

As I explain in more details in the “The arguments behind the initiative” section, given our current state of knowledge, it is difficult to see much benefit from the reform. The arguments behind the reform are inconsistent with much empirical evidence and find little theoretical support. It is typically argued that sovereign money could avoid financial crises. But runs on bank deposits are not the main source of recent crises. The initiative is also based on the surprising idea that money is not a liability. I also discuss the issues with this idea in the “The arguments behind the initiative” section. Finally, the “The arguments behind the initiative” section discusses why bank credit is unlikely to be the source of money creation at the macroeconomic level and relates this view to the “mystique of money.”

Independently of its motivation, the next question is to assess the potential impact of the reform for the Swiss economy. This is done in the “The impact of sovereign money in Switzerland: stage 1” and “The impact of sovereign money in Switzerland: stage 2” sections. The reform is planned to be implemented in two stages. In the first stage, sight deposits, that are part of M1, disappear from bank liabilities and are deposited at the central bank. But the overall banks’ balance sheets may not be affected as the central bank could lend its reserves back to banks. The first stage of the reform and its impact is examined in the “The impact of sovereign money in Switzerland: stage 1” section. In the second stage of the reform, the central bank no longer lends its reserves to banks. This means that banks need to find alternative sources of financing. It also means that the central bank could use its reserves in different ways. These aspects are reviewed in the “The impact of sovereign money in Switzerland: stage 2” section.

The “The impact of sovereign money in Switzerland: stage 1” section examines quantitatively the impact of the reform’s first stage on the state, on banks, and on depositors, using a simple model of monopolistic competition in the banking sector. In the current situation of the Swiss economy, the aggregate impact of the first stage would be negligible because of very low, even negative, interest rates and of a massive level of banks’ reserves at the central bank: in 2017, the proportion of banks’ reserves to deposits in M1 is larger than 90%.

To have an assessment in a period of positive interest rates, I consider data for the 1993–2006 period. I find that the overall impact of the reform is negative and annually represents − 0.4% of GDP. First, seigniorage of the central bank increases, while tax payments by banks decline. Consolidating the SNB and the government, the state gains by 0.7% of GDP. However, depositors would be the main losers (1% of GDP) since they no longer receive a return on their sight deposits. Banks would naturally also lose (0.15% of GDP).

Results in the “The impact of sovereign money in Switzerland: stage 1” section basically represent the impact of imposing full reserve requirement at zero interest rate. But they do not include the impact of the other dimensions of the sovereign money initiative, which are discussed in the “The impact of sovereign money in Switzerland: stage 2” section. The “The impact of sovereign money in Switzerland: stage 2” section reviews the alternative sources of funding for banks in the second stage of the reform. It points to potential instability with some sources of funding. Then, it reviews the implications of a mismatch between SNB’s assets and liabilities. Finally, it discusses the constraints and the dangers for monetary policy.

## The arguments behind the initiative

Several arguments backing the initiative are based on claims that are either incorrect or inconsistent with empirical evidence.

### Mistaken claim 1: credit creates money

A major argument behind the idea of sovereign money is that money creation comes largely from the granting of credit by commercial banks. As a consequence, sovereign money could better control credit. However, there is confusion about this idea and the close relationship between money and credit is not verified at the macroeconomic level.

#### The confusing debate on banks and money creation

It is first useful to mention the debate about the role of banks in the creation of money.^10^ One can distinguish between two perspectives. On the one hand, banks can create deposits when granting loans. This is well explained in textbooks when explaining the money multiplier (even if the money multiplier examples are unrealistic). On the other hand, banks serve as intermediaries between deposits and loans. As explained for example by Tobin (1963), these two perspectives are totally consistent. In equilibrium, the amount of deposits created by banks has to be equal to the amount desired by depositors. And the central bank can influence this equilibrium.

However, there is a group of people that only accepts the first perspective and rejects the second one, which is obviously incorrect (see more on this below).^11^ It is therefore claimed that banks create money “out of thin air” (*aus dem nichts*) and claimed or given the impression that banks can freely decide how many loans and deposits to issue. In this context, the sovereign money supporters find this freedom unacceptable and consequently believe that the central bank should control sight deposits. But this belief is based on the incorrect view of “monetary mysticism.”

Before turning to an example and to empirical evidence to clarify the issue, two comments are worth making at this stage. First, the above discussion only talks about credit and deposits. If these are the only items on banks’ balance sheets, there should be a full correlation between these two variables. But there are other assets and liabilities on commercial banks’ balance sheets, which naturally weakens the link between loans and deposits. For example, loans can be matched by non-deposit liabilities or by a decline in other assets. The second comment is about the Bank of England article by McLeay et al. (2014). Even though this article is supposed to clarify the issues related to money creation, its ambiguous wording is actually creating more confusion. The article is very much in the line of Tobin (1963) that integrates the two perspectives on money creation mentioned above. However, the way the first pages of the article are written initially gives the impression that only banks determine the amount of deposits through their loans. This is the reason why supporters of monetary mysticism cite McLeay et al. (2014) as supporting their view. But this is not the basic message of that article.

#### A simple example

Let us now turn to a simple example. At a microeconomic, partial equilibrium, level, it is true that a bank can increase the quantity of deposits when it provides a loan. But this is only true at the initiation of the loan. Consider a simple example that illustrates why this is not necessarily the case once the loan is being used. Assume I want to buy a house and I ask a mortgage loan from my bank. When my bank grants me the loan, the funds are available on my checking account. So in this initial operation, my bank indeed increases money. Then, I transfer immediately the funds to the seller of the house, who has an account in another bank and will see an increase in her checking account. If the seller keeps the funds in the checking account, her bank can use the funds to make a loan, as it happens in textbook examples of the money multiplier. But assume that the seller does not want to keep these funds in her checking account, as it bears a low interest, and transfers them to interest-bearing instruments of her bank (e.g., time deposits, bank bonds, savings account). Then, at the end of the day, my mortgage loan has no impact on the quantity of checking accounts and on M1, as my loan is matched by an increase in interest-yielding assets of the seller. In other terms, there is no obvious link between credit and sight deposits even when considering a single loan.

It is not clear how things would change under sovereign money. If my bank grants me a loan, the funds still end up in the seller’s checking account and initially increase money. How could the central bank keep money constant if the seller decided to keep the funds on her checking account? The system would need to impose that the new credit is matched by a decline in deposits at the central bank. It is not clear how this constraint would be implemented, but it would most likely be costly and disrupt the efficient allocation of credit. If such constraints are not imposed, it is difficult to see how sovereign money affects the relationship between credit and money.

#### A decoupling between money and credit

**Fig. 1:**
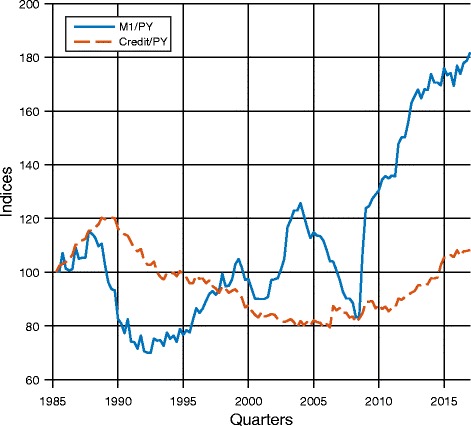
M1 and total credit per GDP. Data source: SNB. Data are from 1985q2 to 2017q1. Both variables are in real terms and then deflated by GDP before being transformed into indices; base 100 = 1985q2. Credit includes mortgages and is total credit issued by Swiss banks to Swiss. This is robust when considering total credit issued by Swiss banks to Swiss and foreigners

As illustrated by the previous example, in general, money is not generated by credit. This is confirmed by macroeconomic data. As mentioned in the Introduction, Schularick and Taylor (2012) document a decoupling between broad money and credit since World War II. This is also true for M1 and credit for Switzerland. Figure 1 shows the evolution of credit and M1 (divided by GDP and normalized to 100 in 1985q2) in Switzerland. It shows that movements in M1 are not tied to movements in bank credit. We see for example that during the credit boom in the early 1990s, M1 actually decreased. Similarly, the large increases in M1 in the second half of the sample are not accompanied by large increases in credit. If we look at the correlation between the changes in money and in credit on a monthly basis from 1985 to 2015, we find a coefficient of − 0.052.^12^

One should also notice that sight deposits represent a relatively small proportion of credit: about 25% in the last decades. In other terms, most of bank credit is not backed by sight deposits in Swiss francs.

#### The constraint of money demand

Claiming that banks create money basically assumes that money demand is totally elastic. In that case, it is the supply that determines the quantity.

But this assumption is not empirically realistic. A standard money market equilibrium can be written as

1 $$ {M}^S=P\cdotp L\left(Y,i-{i}^m,c\right) $$

where *M*^*S*^ is nominal money supply, *P* is the price level, and *L* is a real money demand function from the private sector. It typically depends positively on a measure of economic activity *Y* and negatively on the opportunity cost of holding money *i − i*^*m*^, where *i*^*m*^ is the interest on money and *i* is the alternative interest rate, typically government bonds. The variable *c* represents other factors like financial technology. If we assume that prices are rigid in the short run, an increase in *M*^*S*^ is only possible if *Y* increases or if *i − i*^*m*^ decreases. Since banks cannot directly influence *Y* and *i*, an increase in *M*^*S*^ in the short run can only come from an increase in *i*^*m*^.^13^ Notice that there is one case where money demand is fully elastic. This is the case of a liquidity trap we are currently in. In that case, eq. (1) does not apply as the private sector is indifferent between money and alternative assets.

At the empirical level, there is a very long tradition of estimating money demand.^14^ Even though these estimations are faced with econometric problems, they tend to yield reasonable (and finite) income and interest elasticities. In the Swiss case, the focus has often been on M2 or on M3 as M1 appears less stable and less related to macroeconomic variables like inflation or output.^15^ However, the “The impact of sovereign money in Switzerland: stage 1” section will present a specific estimation for M1.

### Mistaken claim 2: sovereign money avoids financial crises

In theory, a major advantage of a full reserve requirement system or of sovereign money is to avoid traditional bank runs, as modeled in Diamond and Dybvig (1983). This leads the defenders of the initiative to claim that a better control of money would (i) eliminate financial crises, (ii) avoid speculative bubbles, and (iii) avoid the need for a lender of last resort for banks. However, these claims have little basis and are inconsistent with empirical evidence.

#### Bank runs may not be avoided

It is not the case that sovereign money can fully eliminate bank runs, since a run typically comes from liabilities other than sight deposits.^16^ Empirically, Jordá et al. (2017) show that non-deposit bank liabilities, rather than deposits, tend to predict banking crises. Moreover, in the recent global financial crisis, demand deposits by non-financial agents only played a minor role. It is true that the crisis could be viewed in the perspective of runs, i.e., quick withdrawals of funds, as argued in particular by Gorton (2009). However, these runs were not on demand deposits. They started with the asset-backed commercial paper market and then spread to money market funds and other financial institutions.^17^ Commercial banks were not strongly affected by a run on their checking deposits. Even in the case of British bank Northern Rock in 2007, the run came from other financial institutions, i.e., from short-run liabilities that are not included in M1. Moreover, sovereign money may actually facilitate a bank run: if the central bank offers a safe asset, it becomes easier to move out of banking liabilities when there is a decline in confidence in the banking system. To avoid any bank run, the sovereign money reform should add severe restrictions on banks’ other liabilities.

#### Iceland in 2008 as an example

An interesting case is the financial crisis in Iceland in 2008, which is one of the largest observed in history.^18^ The three large banks expanded extraordinarily their balance sheet and their credit in the years before the crisis. In the crisis, they all went bankrupt and were all subject to a run. The main source behind the credit surge and the subsequent withdrawal came from foreign short-term borrowing, as investors were exploiting the interest differential through carry-trade strategies. Controlling M1 in that context would clearly not help.^19^ It may even be counterproductive: restricting M1 would imply a more restrictive monetary policy, which could increase interest rates in Krona. This would make carry trade even more attractive and increase capital flows and credit growth.

It is interesting to notice that, in Switzerland, the only bank that activated the deposit insurance scheme for its depositors in the recent financial crisis was the subsidiary of one of the Iceland banks, Kaupthing.

#### Empirically, money is not a good indicator of financial crises

There is a vast empirical literature studying banking crises and trying to identify the determinants of crises. Different monetary aggregates and different measures of money have been considered (e.g., the level of real money or deviations from trend), but it has proven insignificant. What has proven significant in recent work, however, is credit (e.g., see Gourinchas and Obstfeld 2012 or Schularick and Taylor 2012). There has been much less empirical work on the causes of financial bubbles, but Jordá et al. (2015) show that credit-driven housing bubbles are particularly damaging for the economy.

More generally, periods of strong credit growth are often followed by lower economic activity. Therefore, controlling credit appears to be key for financial stability. This is by now well understood and has been motivating various aspects of financial regulation. But this is not true for money, since the correlation between money and credit is low: controlling money will not necessarily limit credit growth.

#### A lender of last resort is still needed

The recent crisis and other episodes clearly show that when banks run into trouble, it is not due to traditional bank runs. Why would sovereign money affect the role of the state as lender of last resort? Banks may still be “too-big-to-fail”: a bankruptcy may endanger the whole financial system and will affect employment. Other measures of financial regulation are clearly needed to limit the probability of bankruptcy and the need for state intervention.

### Mistaken claim 3: money is not a liability

A major assumption behind the benefits of sovereign money is that money would no longer be a liability of the central bank. And if it is no longer a liability, there is no need to match money with assets and money can then be spent. This view is puzzling, since both in accounting and in monetary economics, money at the central bank (i.e., the monetary base) is always considered as a liability and matched by assets. If money were not a liability and M1 represents for example 100% of GDP, it would mean that the central bank could potentially give away the equivalent of 100% of GDP on top of its usual profits from seigniorage. This would temporarily liberate a substantial amount of resources that could be used in many different ways (e.g., lowering taxes, increasing spending, lowering the debt, subsidizing credit).^20^

#### No reason to change fiscal policies

There are fundamental issues with using central bank assets for fiscal or credit policies. The first issue is that there is no reason why the state should change other aspects of its policies in the case of a monetary reform. This is because sovereign money differs little from debt so that the policies considered are already possible by changing government debt. Changing other policies because of sovereign money would be suboptimal. For example, consider the current situation of a liquidity trap. In standard models, money and debt are actually equivalent in this situation. As a thought exercise, assume that nominal interest rates on government debt are zero for a very long period.^21^ In that case, money and bonds are very similar since no interest has to be paid on either bonds or money. Bonds mature, but they can be rolled over. Therefore, the consolidated state (government + central bank) can issue either bonds or money. This means that if the central bank buys government debt by issuing money, the consolidated state debt position is unaffected. Whatever can be done with money can be done with debt.

#### A central bank needs to hold assets

The second issue is that it is important for a central bank to hold enough assets. There are at least two main reasons for this. First, assets are useful to conduct monetary policy. The central bank may want to be more restrictive and sell its assets to reduce money supply. Or the central bank may want to change the currency or the maturity composition of its assets through foreign exchange interventions or different types of quantitative easing. Not having enough assets may therefore seriously handicap the central bank.

The second reason for the central bank to hold assets is to provide a guarantee for the currency. Currently, banks hold deposits at the central bank because they trust the central bank and because they know that they can withdraw their funds immediately. With sovereign money, deposits at the central bank are not determined by commercial banks and may be less fickle. But reductions in deposits may still occur and may be caused by a decline in trust in the system. If the central bank gets rid of its assets, it will clearly lose credibility and trust in the system may indeed decline.

### Mistaken claim 4: the Benes-Kumhof paper gives support to the Swiss sovereign money reform

The initiative committee cites the working paper by Benes and Kumhof (2012, henceforth BK) and claims that “the IMF confirms the positive impact of the sovereign money reform.” This claim is abusive for three reasons. First, the working paper by BK is simply an academic investigation and is not the official IMF position.^22^ Second, the study is analyzing a reform that is quite different from the initiative submitted to the Swiss people. Some of the key differences between the initiative and the “Chicago plan” experiment in BK are the following: (i) BK consider full reserve requirements and not sovereign money; (ii) BK have only one type of deposits, so that reserve requirement applies to all deposits and not only to sight deposits as in the initiative; (iii) in BK, central bank reserves, and therefore deposits, can yield an interest, while there would be no interest on reserves in the initiative; and (iv) in the second stage of the reform, the central bank would use money to buy back government and mortgage debt in BK. In the initiative, the central bank would distribute the money to the government. Because of these key differences, the impact of the BK experiment are quite different from the initiative.

The third reason why the reference to BK is misleading is that the environment considered does not correspond to important features of the Swiss economy. One feature is that the Swiss economy is currently in a liquidity trap and the existing amount of central bank reserves is already very large. The monetary reform would therefore not increase substantially the reserves at the central bank. Another key feature is that Switzerland is an open economy. This has several implications. First, the real interest rate is strongly influenced by foreign interest rates. Second, banks can easily change their assets and liabilities by changing their positions with non-residents. Third, there is currency substitution and alternative currencies, mainly euros and dollars, can be used for transaction purposes.

All these differences mean that the results from BK are not relevant for the sovereign money initiative. The Chicago plan experiment in BK increases the steady-state level of output by 10% through three channels.^23^ First, there is a large decline in the real interest rate that boosts investment. But the decline in interest rate comes mainly from the debt purchases by the central bank in the second stage of the reform. This aspect is not considered in the initiative. Moreover, the real interest can decline because the model is a closed economy. In an open economy model, this would typically not happen. The second channel is a decrease in distortionary taxes by a large increase in seigniorage (3.6% of GDP). I will explain below that the increase in seigniorage in Switzerland is much lower than that, so that the potential decrease in taxes is limited. On the other hand, by not paying interest on reserves in the sovereign money reform, seigniorage is also very distortionary. I show below that the loss for depositors is larger than the gain for the state. Therefore, the second channel does not appear relevant. The third channel reflects a decline in monitoring costs due to the reduction in credit. But the sovereign money initiative does not foresee a decline in credit. Moreover, the role of monitoring costs in the BK is somewhat odd: it implies by assumption that the smaller the banking sector, the better.

The above discussion therefore shows that the three channels in BK would not apply to the proposed sovereign money reform in Switzerland.

## The impact of sovereign money in Switzerland: stage 1

This section considers the first stage of this reform, where sight deposits are excluded from banks’ balance sheets but where bank funding is unchanged due to loans from the central bank. The focus is on the redistribution of resources among banks, the state, and depositors.^24^ The analysis shows that, not surprisingly, the central bank gains because it does not pay any interest rate on deposits. On the other hand, this lack of interest payment makes depositors lose, as they also have to pay for operational costs. Banks are only affected to the extent that there is more competition with negative return deposits. The impact analysis from this section is actually similar to the case of full reserve requirements. After describing the overall framework in the next subsection, I present the model and the numerical results.

### Overview

The reform implies that all sight deposits are no longer on the balance sheets of commercial banks. This may imply lower funding for banks. If this is the case, in the first stage of the reform, the SNB lends the funds to banks. More specifically, let *H* be the monetary base before the reform, which is made of banknotes and of banks’ reserves at the central bank. With the reform, banks would transfer deposits in M1 to the SNB and are likely to reduce their initial reserves.^25^ This is the quantity of funds that is no longer available to banks for their lending or investment activities. If the SNB lends the equivalent amount to banks, their total resources are unchanged.

Before the reform, the banks’ balance sheet can be written as

2 $$ H\sim +B+L=\tilde{\mathrm{M}1}+S+O+E $$

where *B* is the net asset held by banks (*B* could be negative), *L* is the loan, *S* is the savings deposit, *O* represents other sources of funding, and *E* is the equity.

**Fig. 2:**
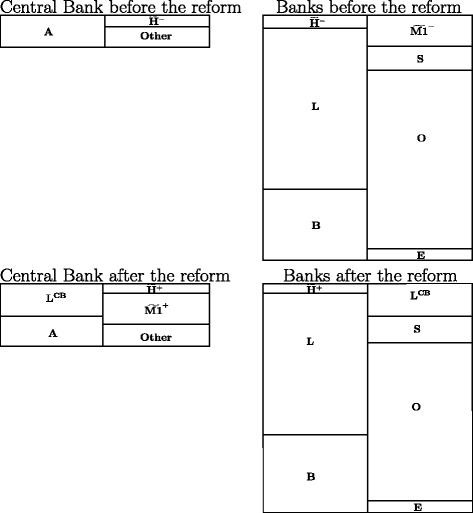
Central banks and commercial banks: prior vs post to the reform. *Notes : A* are total central bank assets, ${\tilde{H}}^{-}\left({\tilde{H}}^{+}\right)$ is monetary base minus banknotes and coins before (after) the reform, *L* are loans issued by banks, *B* are remaining banks assets, ${\tilde{M1}}^{-}\left({\tilde{M1}}^{+}\right)$ is money aggregate M1 minus banknotes and coins before (after) the reform, *S* is savings deposits, *E* is banks capital, *O* are other banks liabilities and *L*^CB^ is the loan the central bank makes to banks after the reform. Areas are proportional using data from December 1996 to June 2017 compiled by the SNB

$\tilde{H}$ is the reserve at the SNB and is equal to *H* minus bills and coins. They yield zero interest rate. $\tilde{\mathrm{M}1}$ represents sight deposits (M1 minus bills and coins). After the reform, $\tilde{\mathrm{M}1}$ disappears from banks’ balance sheets and *H*^−^ is likely to decrease. Banks receive a loan *L*^cb^ from the central bank. This is illustrated in Fig. 2, which shows the commercial banks and the central bank balance sheets. The relative size of the various items in the figure is proportional to their average actual size in the last two decades.

The objective is to analyze the revenue impact for the state, i.e., government and central bank, for banks and for depositors. Three key aspects will influence the analysis. First, an important aspect of the reform is that the SNB would not pay any interest on reserves so that sight deposits would no longer yield any interest. This implies that the opportunity cost of holding money is higher, which has been shown to lower welfare.^26^ Moreover, this will decrease money demand M1. Let m1 represent M1 in proportion of GDP: m1 = *M1/PY*, and let m1^*−*^ and m1^+^ be the levels of money before and after the reform. For the quantitative estimation, it is key to estimate ∆m1 = m1^*−*^ *−* m1^+^. For this purpose, we need an estimate of the interest elasticity of money demand and this is done in the Appendix for the period before the liquidity trap. The important result is the point estimate for interest elasticity of money demand which is *−* 0*.*13. Even though the estimation is derived from a relatively short sample of 22 years, this estimate is in line with the recent estimations of Benati (2016) who considers a sample from 1948 to 2015. This implies that a one percentage point decrease in the interest rate on sight deposits decreases real money demand by 13%. Below, I estimate the decline in the average return on sight deposits to be 2.69. This implies that the reduction in money demand is ∆m1 = *−* 35*.*0%.

**Fig. 3:**
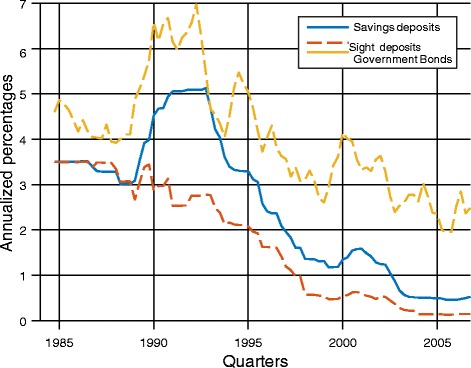
Interest rates, 1984–2006. Data source: SNB. Interest rate on 10-year Government bonds

Second, it is important to distinguish between the current situation of a liquidity trap with interest rates close to zero from a more “normal” situation with positive interest rates. For the more normal period, the estimates will be based on the period 1993–2006. Figure 3 shows the evolution of interest rates during that period. Even though the available data starts in 1984, I start the analysis in 1993 to avoid the high interest rate period of 1989–1992.

Third, the impact of the reform depends on the competitive structure of the banking industry. This is a complex issue, since banks offer multiple products. It is also possible that the competitive structure is affected by the reform. I will abstract from these complexities and follow the macroeconomic literature that assumes monopolistic competition in the loans and the deposit markets.^27^ The next section lays out the underlying model and derives the markups used in the numerical analysis.

### A simple model of monopolistically competitive banks

The model is in line with standard models of banking at the macroeconomic level. Although stylized, this approach allows to determine broadly the magnitude of the effect of the reform. In general, banks offer multiple products in imperfectly competitive markets. To simplify, I assume that there is monopolistic competition with constant markups, generated by Dixit-Stiglitz preferences for deposits and loans. Moreover, the analysis of loans and deposits can be separated as in the Monti-Klein model.^28^

There are four interest rates: *i*^*m*^ on sight deposits, *i*^*s*^ on savings deposit, *i*^*l*^ on loans, and *i* on safe bonds. The safe interest rate *i* is a “reference” rate that applies to government bonds and to the interbank market. I also assume it is the rate at which the SNB would lend to banks.

From profit maximization by monopolistic banks, the difference between the savings interest rate and the bonds interest rate is given by

3 $$ {i}^s=\left(1-{\mu}^d\right)i $$

where *μ*^d^ is the markdown applying to both savings and sight deposits. This abstracts from any cost of managing savings deposits. In the Dixit-Stiglitz framework, the markdown is given by the substitutability across bank deposits and is given by 1 *− μ*^*d*^ = *ε*^*d*^*/*(*ε*^*d*^ *−* 1), where *ε*^*d*^ is the elasticity across bank deposits.^29^ I also assume that there is a proportional cost *c* for banks to run sight deposits.^30^ In that case, the interest rate on sight deposits is given by

4 $$ {i}^m=\left(1-{\mu}^d\right)\left(i-c\right) $$

Banks profits per unit of sight deposits are simply given by ∏ = *i* − (*i*^*m*^ − *c*) = *μ*^*d*^(*i* − *c*).

With sovereign monetary reform, the reference interest rate for banks on sight deposits is zero. Since banks incur a cost *c* in managing sight deposits, I assume that they charge a proportional fee *τ* to depositors. In that case, banks still have an incentive to offer sight deposits. For simplicity, I assume that *τ* = *c*. This neglects the markup of banks in this case, but it is difficult to estimate such a markup when depositors get a negative return. Under this assumption, bank profits on sight deposits are now zero. The decline in return to depositors is equal to *i*^*m*^ + *c*. To estimate the total cost for depositors, the decline in return should be multiplied by the amount of sight deposits.

To quantify the analysis, I consider the 1993–2006 period, avoiding the high interest rate period of 1989–1992. During this period, the average interest rates are *i* = 3*.*32, *i*^*s*^ = 1*.*83, and *i*^*m*^ = 0*.*76. From (3), this implies that 1 *− μ*^*d*^ = 0*.*55 or *μ*^*d*^ = 0*.*45. This implies an elasticity of substitution *ε*^*d*^ = *−* 1*.*23.^31^ From (4), we have *c* = 1*.*93. Therefore, the sovereign money reform implies a decline in the return to depositors of 2*.*69. The decline in bank profits per unit of deposits, *μ*^*d*^(*i − c*), is equal to 0.62.

### Additional revenue for the state

#### Computing additional revenue

A major argument for sovereign money is the increase in revenue for the state. Commercial banks can make a profit by paying a low interest rate on sight deposits and lending the same amount at a higher rate. If instead the central bank controls sight deposits, it can reap these profits. The additional revenue is basically the increase in seigniorage minus two items that are otherwise paid by commercial banks. First, when banks make profits by issuing sight deposits, they pay taxes to the state. With sovereign money these taxes would disappear. Second, there is a cost to manage sight deposits and the liquidity and payment services they provide. At this stage, it is not clear who will pay these costs, but some of these costs may be paid by the central bank. To summarize, the additional revenue from sovereign money can be expressed as^32^

5 $$ \Delta \mathrm{Revenue}=i\cdot \left(\mathrm{m}{1}^{+}-{h}^{-}\right)-{\mathrm{Taxes}}^{-}-{\mathrm{Costs}}^{+} $$

where *h*^*−*^ = *H*^*−*^*/PY*. Equation (5) assumes that banks do not keep any central bank deposits on their balance sheet after the reform.^33^ For convenience, in the numerical analysis, I will abstract from Costs^+^, as they are difficult to estimate. Notice that under sovereign money, the interest differential is simply *i*, because no interest is paid on money. Instead the interest rate differential for commercial banks is *i − i*^*m*^ as they typically pay an interest on money. For an estimation of revenue, we should distinguish between the situation of a liquidity trap that we are in now and more normal times.

#### No increase in revenue in the current liquidity trap

In the current situation, sovereign money would give no additional gain to the central bank. First, interest rates are about zero so that *i* = 0. Moreover, the level of bank reserves is close to the level of demand deposits. These reserves are likely to be mostly replaced by sight deposits. Therefore, sovereign money would increase little the central bank balance sheet and would have no impact on its profits. If the state has to incur some additional costs from managing M1, the net impact could even be negative.

#### Increase in revenue in more normal times

Things will be different if we exit the liquidity trap, where interest rates would be positive, while money demand would be lower. The additional amount of seigniorage with sovereign money will obviously depend on how these variables change. It is natural to assume that the SNB lends its additional resources to commercial banks at rate *i*. If we compute *i ·* (*m*1^+^ *− h*^*−*^) over the period 1993–2006, we find an annual rate of 0.79% of GDP.

To compute the net gain for the state, we need to have an estimate of taxes paid by banks on profits from sight deposit operations. Below, I show that that the decline in bank profits is 0.22% of GDP. If we assume a tax rate of 35%, lost taxes would represent 0.08% of GDP. This implies that the net gain for the state, abstracting from operational costs, would be 0.71% of GDP. Using 2015 GDP, this would make CHF 4.6 billions. This number is not insignificant, but it should be put in perspective by comparing it to recent SNB profits (CHF 24.5 billion in 2016) and to SNB profits that would occur in a period of high interest rates.

### Implications for depositors

In this section, the sovereign money reform has the same implications as a 100% reserve requirement. There is an extensive literature on reserve requirements that shows that they act as a tax on deposits.^34^ With 100% reserve requirement, abstracting from operational costs, the tax is simply equal to the reference interest rate *i* (the marginal interest rate a bank would get if it did not have to hold reserves at the SNB). With perfect competition in banking, this cost would be fully passed through to depositors. In our context of monopolist competition and in the presence of management costs *c*, we saw that the per unit cost is *i*^*m*^ + *c*. The additional tax on depositors from the reform can be simply computed as (*i*^*m*^ + *c*) *·* (*m*1^*−*^ *− h*^*−*^).^35^ For the 1993–2006 period, this gives a loss of 1.00%.

There is an additional cost that we cannot quantify, which is the increase in regulation including likely restrictions for savings deposits. Moreover, there is the uncertainty around these measures.

### Implications for banks’ profits and credit

In normal times, banks would definitely lose from the reform, as sight deposits with cost *i*^*m*^ are replaced by SNB loans with cost *i*. The loss for banks is the decline in interest rate margins, from which we can subtract taxes and operation costs if we assume that they are passed on to depositors. The decrease in interest income is (*i − i*^*m*^ *− c*) *·* (*m*1^*−*^ *− h*^*−*^). Over the 1993–2006 period, this is equal to 0.22% of GDP. If we assume a tax rate of 35% on these profits, the after tax loss in profit would be 0.15%.

Since banks’ balance sheets are little affected by the first stage of the reform and we assume separability between loans and deposits, there is no impact on total credit.

### Overall impact

Table 1 summarizes the above analysis. It is obvious that the precise numbers should be taken with a grain of salt, but the results illustrate the relative gains and losses. An important lesson is that when interest rates are positive, the sum of all the effects is negative. This is due to the decline in *i*^*m*^ with the reform, which implies a decrease in M1.^36^ This decrease means that the gain in SNB revenue is smaller than the loss in net interest revenue from banks. Moreover, the decline in the opportunity cost of holding money is an additional burden to depositors.

**Table 1:** Impact of sovereign money phase 1

Annualized percentage of GDP		
Positive interest rates	Liquidity trap 1993–2006	Current period
SNB	0.79	0
Government	− 0.08	0
State total	0.71	0
Depositors	− 1.00	0
Banks	− 0.15	0
Total	− 0.44	0

Notes: See text for a description. Does not include cost to borrowers, additional costs for the SNB, or regulation costs

To summarize this section, we have found that in the current situation of a liquidity trap, there would be little aggregate impact of the first stage of the reform. If the Swiss economy returns to positive interest rates, the impact would be more significant. Using data for the period 1993–2006, we see not only an increase in state revenue but also a loss for depositors. The loss to banks appears relatively small. Overall, this implies a net loss for the economy. This loss should be seen as a lower bound, as it excludes some of the costs that are more difficult to assess (regulation costs, implementation costs) and it assumes an orderly implementation of the reform.

## The impact of sovereign money in Switzerland: stage 2

In the second stage of the reform, the SNB eliminates its lending to banks. This means that banks need to look for alternative sources of funds. On the other side, the SNB has more potential resources that could be used in several ways. This section will discuss the macroeconomic implications of this second phase under different scenarios. In such a survey, only the broad implications are considered. A more detailed analysis would require a full dynamic model.^37^

### Need for alternative funding by banks

On average, sight deposits represent a relatively small share of banks’ balance sheets. In the last 30 years, sight deposits minus reserves at the central bank represented about 25% of total credit and 15% of total banks’ balance sheets. In the second phase of sovereign money, banks would need to find alternative sources of funding. Given the attractiveness of the Swiss franc, there is no doubt that Swiss banks would be able to find funding, at least for large banks. However, switching to alternative funding may create short-term costs. For example, consider the situation where banks want to rapidly increase their credit and need to issue new liabilities. Such a situation would occur if the Swiss economy exits the liquidity trap. In the transition, it might take some time to organize alternative funding, especially for smaller banks. This may slow down a potential credit recovery. Therefore, there might be short-run risks in the search for alternative financing.

In the medium run, the question is whether this funding would be much more expensive than sight deposits. This is a difficult question. Sight deposits obviously imply a lower interest payment for banks. But a large part of the lower interest rate is accounted for by the operating cost of sight deposits. Therefore, the difference may not be that large.

What type of alternative funding would be available? The basic idea behind the initiative is that, once sight deposits are outside of banks’ balance sheets, the financing of banks should come from more “responsible” investment decisions. This is likely to be true for equity or long-term debt. But some alternative sources of financing may not be more “responsible” and some other may make banks more prone to crises. First, there might be an increase in savings deposits: since the opportunity cost of holding sight deposits increases, there would be a shift towards savings deposits. Second, there might be a shift towards sight deposits in euros. These deposits would not be part of sovereign money and would keep yielding a positive interest rate (once we exit the current liquidity trap). These accounts are already available in many Swiss banks, so that the switch would be easy. It may lead to an increase in euro transactions in Switzerland.^38^ Third, banks may innovate to make alternative investments more liquid (e.g., the citation of Cochrane in the “Background” section). Basically, they can reduce switching costs between invested funds and money needed for transactions. This could drastically reduce the demand for sight deposits without changing the behavior of depositors.

But alternative funding may attract more fickle funding. For example, banks may rely on short-term debt borrowing from other financial institutions. These sources of funds are more volatile than sight deposits, as the recent financial crisis has illustrated (e.g., Bear Stearns, Lehman Brothers, or Northern Rock). There are many other examples of dramatic financial crises, where the source of the problem is the short-term international borrowing by banks and not in demand deposits (e.g., the Asian crisis or Iceland).^39^ In particular, this could increase the exposure of Swiss banks to international contagion. In other terms, the Swiss banking system may replace funding from relatively stable funding deposits by funding from more volatile sources and be more prone to financial crises. Moreover, by offering a safe asset outside the banking sector, sovereign money makes it easier for these funds to leave banks.

Even outside of financial crises, a more volatile source of funds may affect banks’ lending behavior. For example, Paligorova and Santos (2017) show that banks that rely more on wholesale funding than on insured deposits have a shorter maturity of loans and that longer maturity loans are more expensive.

### Macroeconomic implications

The macroeconomic impact of the reform depends on how the additional money at the SNB is used. For example, Benes and Kumhof (2012) assume that the state buys back mortgage and government debt, which leads to a decline in the interest rate and an increase in investment. Mortgage buybacks are not considered by the initiative, and I will focus on more realistic scenarios.

#### Status quo

The SNB invests its resources in Swiss and foreign assets. This could still be the case with sovereign money if additional money is simply matched by increases in SNB assets. SNB profits would come, as now, from the return differential between its assets and reserves. These profits would then be distributed over time to the state. The impact of sovereign money would not be large, besides the negative net effect mentioned in the previous section.

#### Increased transfers from the SNB

However, the initiative would insert in the Swiss constitution that the new money created by the SNB is directly transferred to the state (cantons and confederation) or to the private sector. This would also apply to the existing stock of $\tilde{\mathrm{M}1}$. The initiative committee argues that the SNB could transfer an additional CHF 300 billion to the state, based on the size of M1 a decade ago (even though $\tilde{\mathrm{M}1}$ never reached that amount before 2009). But the size of M1 has more than doubled (to reach about 100% of GDP), and if the SNB had to distribute the equivalent of $\tilde{\mathrm{M}1}$, this would be a colossal amount. It might also have to sell most of its assets (which would put huge pressure on the Swiss franc) in the likely case that banks drastically reduce their central bank deposits in their balance sheet, $\tilde{H}$. To avoid an initial sale of assets, the SNB could only distribute $\tilde{\mathrm{M}1}-\Delta \tilde{H}$. In the case where banks were to eliminate their central bank reserves from their balance sheet, this would amount to CHF 55 billion, which is a much smaller amount.^40^ In any case, there seems to be uncertainty about the extent of distribution by the SNB. However, the precise amount distributed would have a negligible impact on the present value of SNB transfers: SNB profits are anyway eventually distributed to the state. In other words, the initiative’s committee is basically proposing to frontload the distribution of SNB profits at the cost of lower profits for future generations.

Nevertheless, policies affecting the timing of transfers may have distortionary effects. The actual impact of these transfers depends on what the state would do. If central bank transfers are exclusively used to reduce government debt, the impact is likely to be small. This would not affect government expenditures or revenues and would leave unchanged the consolidated position between the state and the central bank. However, it would also reduce the size of Swiss public debt, which may not be desirable.^41^

If the SNB transfers the increase in money directly to the private sector, this would be equivalent to “helicopter money” (a policy where the central bank makes direct transfers to the private sector). Such a policy is currently discussed in the context of the liquidity trap but is clearly not the right policy in normal times for reasons I will not discuss here.

A more likely scenario is that these transfers will allow to finance government deficits, i.e., to increase its expenditures or to decrease its revenues without a need to issue debt. This means that monetary policy would be tied to fiscal policy. It is well known that deficit financing by the central bank is extremely bad policy. All modern central banks are prevented from directly financing the government, and the SNB has always been a leading example in terms of independence. It would also be important that central bank transfers affect fiscal policy as little as possible. Putting the emphasis on a frontloaded distribution of central bank profits may help in “selling” the initiative to the voters but is not key to a monetary reform. Moreover, it would clearly put political pressure on the SNB.

### Implications for monetary policy

Monetary policy would clearly be hampered by the sovereign money initiative. In the ideal world of a smoothly growing economy, the SNB could gradually increase its money supply through transfers (with all the problems this entails). But in the real world, the economy is bumpy and the SNB needs to react quickly to the changing economic environment. With the initiative, the SNB may no longer be able to use its current instruments that work in great part through a quick impact on the monetary base. As already mentioned, it is not clear how many reserves banks would still hold at the central bank, since they would no longer have to face liquidity shocks from demand deposits. The SNB may have to find other, less efficient, ways to influence monetary policy. In particular, it is not obvious to foresee how the SNB would operate when monetary policy has to become more restrictive for a sustained period.^42^ Following the logic of the initiative, to reduce M1, the SNB should do reverse transfers to the government, i.e., tax the government. This appears unrealistic and extremely difficult to implement politically. An alternative could be to issue central bank bills to reduce money supply. But how safe would central bank debt be perceived if its assets do not match existing liabilities? Investors may require a high risk premium to hold these bills, which would make monetary policy very costly. Moreover, once there is central bank debt, could it be reduced to increase again money supply? This might contradict the law, as money supply increases are supposed to be transferred to the state or to the public.

Another issue for monetary policy is that the initiative implies that the SNB would return to monetary targeting, since it focuses on money supply. The SNB adopted such a strategy after the end of the Bretton Woods system until 2000 when it shifted to a policy focusing on inflation forecasts and on the control of short-term interest rates. There were good reasons (which I will not review here) to abandon such a system, and going back to it would clearly lead to worse monetary policy. More generally, setting constraints in the federal constitution on the way monetary policy can be implemented is undesirable and inconsistent with central bank independence.

## Conclusions

This survey has evaluated the arguments behind the sovereign money initiative and has examined some of its potential consequences. This has been done from a monetary and macroeconomic perspective and the survey abstracts from important aspects related to legal issues, practical implementation, or implications for specific institutions. One element that has been mentioned, but could not be evaluated, is uncertainty. There is high uncertainty at two levels. First, the text of the initiative is not precise and there is uncertainty about how it could be implemented. Second, since such a system has never been implement anywhere, there is high uncertainty about the reaction of economic agents. For example, one scenario could be that the initiative would stimulate financial innovation and that financial technology would allow to make payments without any sight deposits in Swiss francs. Trying to guess which scenario is the most likely is difficult, but what is clear is that this high uncertainty would be an additional cost from this initiative.

This survey puts the initiative in a negative light, as its foundations are shaky, its benefits are questionable, and its drawbacks can be serious. Before starting working on the survey, I had a much more positive prior. However, the more I delved into the issue, the more disappointed I became because of the limited intellectual merit in the arguments behind the monetary reform proposal. First, it ignores and even despises current knowledge in monetary economics. Several of the arguments made are inconsistent with this knowledge and with basic economic logic. For example, claiming that bank credit creates money is inconsistent with empirical evidence and there is no convincing argument that sovereign money can avoid financial crises. Second, some of the claims are misleading or demagogic. For example, it is not true that the IMF supports the initiative or that there is academic support for it.

A major theme in this paper is that the role of sight deposits is overstated in the arguments behind the initiative. There is no evidence, at least in the last 80 years, that increases in sight deposits would lead to financial crises or to large credit increases. Therefore, giving control of these deposits to the SNB cannot provide any stabilizing benefit. On the other hand, the sovereign money reform will entail clear costs for the Swiss economy and will create potential risks and instability. The quantitative analysis shows that depositors would clearly lose from the reform and that these losses are larger than the increase in state revenue. Pushing banks to look for alternatives to sight deposits is potentially destabilizing. There is a clear destabilizing impact of the reform, even though it is difficult to evaluate this quantitatively. Creating a SNB balance sheet mismatch and constraining monetary policy are threats to monetary stability and to the well functioning of the Swiss economy. It is to be hoped that all these costs and potential risks will be well understood by Swiss voters.

## Appendix: interest elasticity of money demand

The objective is to determine how much the demand for sight deposits would decrease with a decline in its interest rate. This amounts to estimate a semielasticity of money demand: by how much, in percent, does money demand decrease if the interest rate increases by one percentage point? Estimates of this elasticity vary a lot, from as low as 6 in Ireland (2009) to as high as 60 in Bilson (1978).^43^ Here, we estimate a simple long-run money demand for Switzerland, using quarterly data from 1984q4 to 2006q4. Our interval ends in 2006 to focus on a period where interest rates were distinctly higher than zero. As a dependent variable, we consider deposits in M1, so that we subtract banknotes from M1 and define this new variable as estimate of the following regression:

A1 $$ \ln\;\left({\tilde{\mathrm{M}1}}_t/{P}_t\right)={\alpha}_0+{\alpha}_1\;\ln {Y}_t+{\alpha}_2\left({i}_t-{i}_t^m\right)+{u}_t $$

where *P*_*t*_ represents the consumer price index, *Y*_*t*_ is the real GDP, *i*_*t*_ is the long-run interest rate (10-year Swiss bonds), and *i*^*m*^ is the interest rate on sight deposits. The important result below is the point estimate for *α*_2_, which is *−* 0*.*13.

### Data

Data is quarterly for the period 1984q4–2006q4 and comes from the SNB database. Monthly variables were converted to quarterly using the end of quarter value. Money aggregate M1, banknotes, and nominal GDP are in billion CHF. The interest rate differential is calculated as the difference between the long-run interest rate on bonds (10-year confederation) and the interest rate on sight deposits. Both rates are annualized and in percentage points. *P*_*t*_ is CPI based on all items (base 100, 2015m12).

### Regression

The regression performed is as follows:

$$ \ln \left(\frac{\left(\mathrm{M}1-\mathrm{banknotes}\right)t}{P_t}\right)={\alpha}_0+{\alpha}_1\;\ln \left(\frac{{\mathrm{GDP}}_t}{P_t}\right)+{\alpha}_2\left({i}_t-{i}_t^m\right)+{u}_t $$ (A2)

Results are displayed in Table 2.

**Table 2:** Demand for sight deposits

Variables	Coefficient	Std errors	Robust std errors	*T* test	*P* value
Constant	− 18.54	0.01	1.52	− 12.23	0.00
ln(Real GDP)	3.72	0.00	0.22	16.98	0.00
*i − i* ^*m*^	− 0.13	0.01	0.02	− 8.19	0.00

Notes: Dependent variable is ln $\left(\frac{\left(\mathrm{M}1-\mathrm{banknotes}\right)}{\mathrm{CPI}}\right)$ Adjusted *R*^2^ = 0.80062. Durbin-Watson = 0.53708

Notice that the dependent variable and the exogenous variable ln $\left(\frac{{\mathrm{GDP}}_t}{P_t}\right)$ are both *I*(1), so that we have to check for cointegration. Residuals of the regression are stationary according to the Augmented Dickey-Fuller test. Moreover, in an error correction model, the error correction term is significant.

## Footnotes

1 In Swiss national languages, sovereign money is called *Vollgeld*, *monnaie pleine*, or *moneta intera*. It is useful to consider both the text of the initiative requiring a change in the Swiss constitution and its interpretation by the Swiss Federal Council (see www.admin.ch/gov/en/start/documentation/media-releases.msg-id-64444.html). The website of the initiative committee is www.vollgeld-initiative.ch.

2 This paper is brief on some aspects that are already covered in details elsewhere, e.g., see the views of the Federal Council mentioned in footnote 1 or *Die Volkwirtschaft*, Nr. 1–2, 2017, pp. 47–55.

3 For example, see Huber (2015, p. 15) for a discussion of the difference between full money and 100% reserves. With full reserve banking, deposits remain on the banks’ balance sheets, while they are off-balance sheet with sovereign money.

4 For recent contributions, see Benes and Kumhof (2012), Baeriswyl (2014), Cochrane (2014), or Prescott and Wessel (2016). See Benes and Kumhof (2012, section III) for a review of the Chicago plan.

5 The initiative committee mentions a KPMG (2016) report as major source of literature. This report reviews the Benes and Kumhof paper as well as 13 other studies analyzing the costs and benefits of full reserve requirements or of sovereign money. Some of these studies are actually quite critical with the elements of sovereign money reform. Moreover, none of these studies focuses on the Swiss initiative or the Swiss economy.

6 See also Huber (2014) and other writings by this author.

7 Notice that most of the literature does not make a distinction among different monetary aggregates. In the same spirit, this introduction simply talks about money, but the rest of the paper will be more precise in focusing on M1.

8 Cochrane (2014, p. 199) puts it clearly: “With today’s technology, you could buy a cup of coffee by swiping a card or tapping a cell phone, selling two dollars and fifty cents of an S&P 500 fund, and crediting the coffee seller’s two dollars and fifty cents mortgage-backed security fund. If money (reserves) are involved at all—if the transaction is not simply netted among intermediaries—reserves are held for milliseconds. In the 1930s, this was not possible.”

9 An interesting case is the experience of Ecuador where e-money is issued by the central bank but only receives limited public acceptance.

10 This debate has been particularly active in recent years on the blogosphere. For example, there have been numerous reactions to a series of blogs published by Paul Krugman.

11 They also argue that most economists do not understand how banks work, since in their models, they tend to focus on the second perspective. In his blogs, Krugman calls this view “monetary mysticism” or “banking mysticism” and is related to the “mystique of ‘money’” in Tobin (1963).

12 The correlation is also insignificant if we consider M2 or M3.

13 Banks could obviously have an indirect effect. For example, an increase in credit could boost economic activity, which stimulates money demand.

14 There has been declining attention to money demand in the last two decades as central banks focused more on inflation targeting and decreased their focus on monetary aggregates.

15 For example, see Kirchgässner and Wolters (2010).

16 There could also be a run on the asset side, as customers may run down their credit lines in times of crises. See Ippolito et al. (2016) for evidence.

17 Gorton (2009) writes: “today’s panic is not a banking panic in the sense that the traditional banking system was not initially at the forefront of the “bank” run as in 1907... In the current case, the run started on off-balance sheet vehicles and led to a general sudden drying up of liquidity in the repo market, and a scramble for cash...”

18 See for example Benediktsdottir et al. (2011) for a description of the Iceland crisis.

19 The proposal of sovereign money has also been suggested in a report by Sigurjónsson (2015), but the report does not explain how the financial crisis could have been avoided.

20 If this view were true, one could wonder why countries would not have already used the resources.

21 See Bacchetta et al. (2016) for a formal analysis of a persistent liquidity trap.

22 On the first page, it is written: “This Working Paper should not be reported as rep- resenting the views of the IMF. The views expressed in this Working Paper are those of the author(s) and do not necessarily represent those of the IMF or IMF policy. Working Papers describe research in progress by the author(s) and are published to elicit comments and to further debate.”

23 Another issue is that the BK model is not standard and incorporates several debatable assumptions. It is also difficult to see the role of each assumption on the results. A more detailed discussion of these issues would become quite technical for this survey.

24 For a welfare analysis of different scenarios, see Bacchetta and Perazzi (2017).

25 There is uncertainty as to how many reserves banks would still need if they no longer have demand deposits on their balance sheet.

26 For example, see Curdia and Woodford (2011). This point is related to the Friedman rule, a basic result in monetary economics. It says that the optimal level of nominal interest rates on bonds should be zero to eliminate the cost of holding money (when money yields zero interest). Since bonds rates are usually positive, it is optimal to pay a positive interest rate on money.

27 Obviously, a simplified macroeconomic model does not deal with microeconomic aspects that may turn out to be significant. For example, there is evidence that sight deposits provide useful information to banks and improves loan monitoring (see Mester et al. (2007). A decline in sight deposits would decrease this information value.

28 See, for example, Generali et al. (2010) for similar set of assumptions in a DSGE model.

29 Notice that this elasticity is different from *α*_2_, which represents the elasticity between sight deposits and other assets.

30 For simplicity, I assume that there are only variable costs, even though in reality fixed costs are significant.

31 Notice that this estimated elasticity is relatively low. This is explained in part by the assumption of no operational cost for savings deposits.

32 There are two ways to look at seigniorage. First, a central bank earns revenues by issuing money at a low or zero interest rate and lending it at a higher interest rate. In that case, seigniorage is equal to the interest differential times the stock of money. In the second perspective, seigniorage is simply the money created by the central bank. Although the two perspectives appear different, under some mild conditions, they turn out to be equivalent in present value. We focus on the first approach.

33 This has a negligible impact on the numerical results as *h* is very small (less than 1% of GDP) in the sample under consideration, i.e., before the global financial crisis.

34 Under some conditions, reserve requirements are equal to a tax on deposits combined with an open market operation. See Bacchetta and Caminal (1994).

35 It can be argued that the tax should be computed on the new money demand, i.e., *i*^*s*^ *·* (*m*1^+^ *− h*^*−*^). However, when m1 decreases, depositors enjoy fewer services from sight deposits or have to incur higher costs. A simple approximation of these costs is (*i*^*m*^ + *c*) ∆m1 and is captured by using m1^*−*^ instead of m1^+^. The precise measure of these costs actually depends on the motives for holding money. For an analysis of the welfare cost for depositors in a more structured analysis, see Bacchetta and Caminal (1992).

36 Notice that the decrease in M1 is computed by using the interest elasticity of money demand before the reform. It could be that new financial products are offered after the reform so that this elasticity increases. With a higher interest rate elasticity, both the state revenues and the loss for depositors would be smaller.

37 Bacchetta and Perazzi (2017) analyze various scenarios in a dynamic model, examining in particular the welfare effect of a reform.

38 Notice that almost half of Swiss banks’ liabilities are already in foreign currency. However, an increase in foreign currency liabilities could imply a currency mismatch for Swiss banks.

39 As already mentioned, Jordá et al. (2017) show that non-deposit sources of funding increase the probability of financial crises.

40 Notice that even with this smaller initial transfer, the SNB may be forced to sell faster its existing foreign assets. As the Swiss economy exits the liquidity trap, sight deposits are likely to substantially decrease (as the nominal interest rate on other assets increases) and the SNB would need to sell its existing foreign currency assets. If this is preceded by a large distribution of funds, the SNB will be left with a smaller stock of assets.

41 See Bacchetta (2017) for a discussion. Notice also that the stock of Swiss public debt is currently much smaller than M1, so there might not be enough debt to buy.

42 This is less problematic in stage 1 of the reform since the SNB would lend a large amount to banks. It could then restrict the amount of lending.
